# Assessment of Mental Health of Chinese Primary School Students Before and After School Closing and Opening During the COVID-19 Pandemic

**DOI:** 10.1001/jamanetworkopen.2020.21482

**Published:** 2020-09-11

**Authors:** Lei Zhang, Dandan Zhang, Jiao Fang, Yuhui Wan, Fangbiao Tao, Ying Sun

**Affiliations:** 1 Department of Maternal, Child & Adolescent Health, School of Public Health, Anhui Medical University, Hefei, Anhui Province, China; 2 Key Laboratory of Population Health Across Life Cycle (Anhui Medical University), Ministry of Education of the People’s Republic of China, Hefei, Anhui, China; 3 Anhui Provincial Key Laboratory of Population Health and Aristogenics, Anhui Medical University, Hefei, Anhui, China

## Abstract

The cohort study assesses the association of school closings during the COVID-19 pandemic with the mental health of primary school students in China.

## Introduction

During the coronavirus disease 2019 (COVID-19) pandemic, most governments across the globe have temporarily closed schools, a decision that has impacted 1.4 billion students worldwide.^1^ The outbreak of COVID-19 took place during the winter vacation (originally scheduled during January 21 to February 19, 2020) of Chinese schools while all the students were at home. On January 27, China’s Ministry of Education announced that the 2020 spring semester for schools would be postponed to late April owing to the novel coronavirus outbreak, affecting 278 million students across primary and postsecondary grades in China. Although recent modeling studies predict that school closures alone would prevent only 2% to 4% of deaths,^2^ school closures may be associated with mental health problems among students owing to a prolonged state of physical isolation from peers, teachers, extended family, and community networks.^3,4^ Most of the available data are cross-sectional, and not much is known about the long-term mental health outcomes associated with prolonged school closure among children and adolescents.^5^ This longitudinal cohort study investigated psychological symptoms, nonsuicidal self-injury, and suicidal ideation, plans, and attempts among a cohort of children and adolescents before the outbreak started (wave 1, early November 2019) and 2 weeks after school reopening (wave 2, mid-May 2020) in an area of China with low risk of COVID-19.

## Methods

This cohort study is part of an ongoing longitudinal study on physical and mental health factors associated with early adversity among children in China. Students in grades 4 through 8 from local primary and junior high schools in 2 counties of Chizhou, Anhui Province, were invited to participate in the study during early November 2019. A total of 1389 children were recruited, and 1271 (95.3%) of them had complete information. After 3 months of lockdown, schools in Chizhou were reopened on April 26. Of the original 1389 students in the wave 1 survey, 1333 participated in the follow-up questionnaire survey in mid-May 2020 (wave 2). A total of 1241 completed questionnaires were received with a response rate of 93.1%. Ethical approval was obtained from Anhui Medical University. Written informed consent was provided from both eligible children and their guardians as well as school administrators. This study followed the American Association for Public Opinion Research (AAPOR) reporting guideline.

Data on depressive and anxious symptoms, nonsuicidal self-injury, suicide ideation, suicide plan, and suicide attempt were collected in 2 waves. Detailed information on mental health outcomes measurement is given in the eMethods in the Supplement.

Proportions and χ^2^ tests documented differences in the prevalence of mental health problems between the 2 waves. Binomial family generalized estimating equations with a logit link were used to test for trends in the prevalence of psychological symptoms, nonsuicidal self-injury, and suicidal behaviors changes. The model included age and wave of survey as independent variables adjusting for sex, body mass index, self-perceived household economic status, family cohesion, parental conflict, academic stress, parental educational level, family adverse life events, self-perceived health, sleep duration, and sleep disorders. Analyses were performed using Stata/SE, version 13.1 (StataCorp LLC). Results were evaluated at *P* < .05 (2-tailed). Data were analyzed from May 22, 2020, to June 12, 2020.

## Results

A total of 1241 students participated in the 2 waves of the survey (mean [SD] age, 12.6 [1.4] years; age range, 9.3-15.9 years; 736 [59.3%] male). A total of 729 fathers (58.7%) had a lower secondary education level, and 325 (26.2%) and 187 (15.1%) had higher secondary and tertiary levels, respectively. A total of 102 children (8.2%) resided in households with a monthly disposable income of US $288 or fewer. As shown in the Figure and Table, the prevalence of mental health outcomes among students in wave 2 increased significantly from levels at wave 1: depressive symptoms (24.9% [309 of 1241] vs 18.5% [235 of 1271]; adjusted odds ratio [aOR], 1.50 [95% CI, 1.18-1.90]; *P* = .001), nonsuicidal self-injury (42.0% [521 f o1241] vs 31.8% [404 of 1271]; aOR, 1.35 [95% CI, 1.17-1.55]; *P* < .001), suicide ideation (29.7% [369 of 1241] vs 22.5% [286 of 1271]; aOR, 1.32 [95% CI, 1.08-1.62]; *P* = .008), suicide plan (14.6% [181 of 1241] vs 8.7% [110 of 1271]; aOR, 1.71 [95% CI, 1.31-2.24]; *P* < .001), and suicide attempt (6.4% [79 of 1241] vs 3.0% [38 of 1271]; aOR, 1.74 [95% CI, 1.14-2.67]; *P* < .001). However, no similar increases in anxious symptoms were found between the 2 waves (15.9% [197 of 1241] vs 13.5% [171 of 1271]; aOR, 0.77 [95% CI, 0.58-1.03]; *P* = .09).

**Figure.**
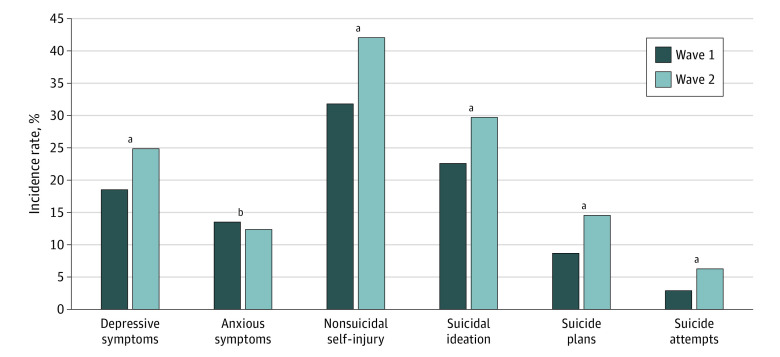
Comparison of the Incidence of Mental Health and Suicidal Behaviors Between the Wave 1 and Wave 2 Groups Wave 1 was before the outbreak started (early November 2019), and wave 2 was 2 weeks after school reopening (mid-May 2020). ^a^*P* < .001. ^b^*P* = .09.

**Table.** Unadjusted and Adjusted Odds Ratios of Depressive Symptoms, Anxious Symptoms, Nonsuicidal Self-injury, and Suicidal Behaviors by Time of Survey

Mental health outcome	Odds ratio (95% CI)^a^	
	Unadjusted	Adjusted^b^
Symptom		
Depressive	1.46 (1.27-1.67)	1.50 (1.18-1.90)
Anxious	1.21 (1.02-1.44)	0.77 (0.58-1.03)
Nonsuicidal self-injury^c^	1.55 (1.40-1.72)	1.35 (1.17-1.55)
Suicide^c^		
Ideation	1.45 (1.27-1.67)	1.32 (1.08-1.62)
Plan	1.80 (1.47-2.21)	1.71 (1.31-2.24)
Attempt	2.20 (1.56-3.10)	1.74 (1.14-2.67)

^a^ Generalized estimating equations models with logit-link function were used. Odds ratios are given for results at wave 2 compared with wave 1 (reference).

^b^ Adjusted for age, sex, body mass index, parental educational level, household economic status, family adverse life events, self-perceived health, academic stress, family cohesion, family conflict, sleep duration at follow-up, and sleep disorder at follow-up.

^c^ Additionally adjusted for depressive symptoms.

## Discussion

These findings highlight mental health effects associated with lengthy school closure owing to the COVID-19 lockdown among children and adolescents in China, which might help to inform other regions affected by COVID-19 on how to timely prepare for the potential increase in mental health problems among children and adolescents returning to school. The preliminary findings were consistent with a recent systematic review^3^ suggesting the association between enforced social isolation imposed by disease containment measures with future mental health problems among children and adolescents. This study has several limitations, including measurement errors in mental health outcomes and unmeasured confounders, as well as the limited representativeness of the sample. Because suicide is the third leading cause of death for children and adolescents aged 10 to 14 years, a wide-ranging interdisciplinary response that applies knowledge about effective suicide prevention approaches should be an international priority.

## Notes

Supplement. **eMethods**

## References

[1] UNESCO COVID-19 educational disruption and response. Accessed June 10, 2020. https://en.unesco.org/news/covid-19-educational-disruption-and-response

[2] Viner RM, Russell SJ, Croker H, School closure and management practices during coronavirus outbreaks including COVID-19: a rapid systematic review. Lancet Child Adolesc Health. 2020;4(5):397-404. doi:10.1016/S2352-4642(20)30095-X 32272089 PMC7270629

[3] Loades ME, Chatburn E, Higson-Sweeney N, Rapid systematic review: the impact of social isolation and loneliness on the mental health of children and adolescents in the context of COVID-19. J Am Acad Child Adolesc Psychiatry. 2020;S0890-8567(20)30337-3. doi:10.1016/j.jaac.2020.05.009 PMC7267797 32504808

[4] Xie X, Xue Q, Zhou Y, Mental health status among children in home confinement during the coronavirus disease 2019 outbreak in Hubei Province, China. JAMA Pediatr. Published online April 24, 2020. doi:10.1001/jamapediatrics.2020.1619 32329784 PMC7182958

[5] Lee J Mental health effects of school closures during COVID-19. Lancet Child Adolesc Health. 2020;4(6):421. doi:10.1016/S2352-4642(20)30109-7 32302537 PMC7156240

