# Supplementary material for: Assessment of Mental Health of Chinese Primary School Students Before and After School Closing and Opening During the COVID-19 Pandemic
Source: JAMA Netw Open. 2020 Sep 11;3(9):e2021482. doi: 10.1001/jamanetworkopen.2020.21482 (PMC7489803; doi:10.1001/jamanetworkopen.2020.21482)
Supplement: Supplement. — eMethods [file jamanetwopen-e2021482-s001.pdf]

Supplementary Online Content

Zhang L, Zhang D, Fang J, Wan Y, Tao F, Sun Y. Assessment of mental health of Chinese primary school students before and after school closing and opening during the COVID-19 pandemic. *JAMA Netw Open*. 2020;3(9):e2021482.
doi:10.1001/jamanetworkopen.2020.21482

eMethods.

This supplementary material has been provided by the authors to give readers additional information about their work.

© 2020 Zhang L et al. *JAMA Network Open.*

Supplementary eMethods

Depressive symptoms

Depressive symptoms were assessed at baseline and follow-up using the long version of the child self-report Mood and Feelings Questionnaire (MFQ) (Costello & Angold, 1988) with a score of 0 indicating no depressive symptoms and a score of 66 indicating very high depressive symptoms. A score of ⩾27 was used as a cut-off score for depressive symptoms. This cut-off score has been shown to give the best diagnostic confidence with good sensitivity and specificity. The split-half reliability was 0.90, the Cronbach α was 0.93, and the test-retest reliability was 0.84 in Chinese children and adolescents (Cao et al., 2009).

Anxious symptoms

Anxious symptoms (generalized anxiety) were reported by adolescents using the MacArthur Health & Behavior Questionnaire (Boyce et al., 2002) for Late Childhood and Adolescence (9-18 years). Responses to 12 item, eg. 'worries about things in the future, "nervous, high strung, or tense", 'physical problems without known medical cause', 'needs to be told over and over that things are OK', are scored on a 3-point scale consisting of 0 ("rarely applies"), 1 ("applies somewhat"), and 2 ("certainly applies"). The score was obtained by computing the mean of the item scores of the dimension. The 85th percentile at baseline (score 4.17 or more) was used as the cut-off point to identify those students with anxious symptoms. The HBQ shows strong internal consistency within our sample, with high internal consistency Cronbach α for anxious subscale (0.831).

Non-suicidal self-injury (NSSI)

At baseline and follow-up, all participants received a screening questionnaire for NSSI, asking 'In the past 12 months, have you ever harmed yourself in a way that was deliberate, but not intended to take your life?'. A list of eight NSSI methods were specified: hit yourself, pulled your own hair, banged your head or fist against something, pinched or scratched yourself, bitten yourself, cut or pierced yourself and burned yourself. Participants were then asked, 'Have you ever done something with the intention of hurting yourself other than what was presented?' For those who confirmed that they had engaged in NSSI, the frequency of NSSI was investigated. NSSI was dichotomised (frequency of NSSI of three or more versus fewer than three as yes or no, respectively) for analysis. The internal consistency reliability of NSSI was 0.780 in Chinese adolescents (Wan et al., 2015).

Suicide ideation, suicide plans and suicide attempts

At baseline and follow-up, suicidal ideation, suicide plans and suicide attempt were assessed, referring to the 'middle school questionnaire' of the 2013 Youth Risk Behaviour Surveillance System in the USA (Centers for Disease Control and Prevention, 2013; Wan et al., 2019). Suicidal ideation was defined as a 'yes' in response to the question 'Have you ever thought about killing yourself in the past 3 months?'. Suicide plan was defined as a 'yes' in response to the question "During the past 3 months, did you make any plans to kill yourself?" Suicide attempt was defined as a 'yes' in response to the question 'Have you ever tried to kill yourself in the past 3 months?'.

© 2020 Zhang L et al. *JAMA Network Open.*

References

1. Cao FL, Su LY, Cheng PX. Reliability and Validity of the Mood and Feelings Questionnaire in Chinese Adolescents. *Chin J Clin Psychol*. 2009; 17(4):440-442.
2. Costello EJ, Angold A. Scales to assess child and adolescent depression: checklists, screens, and nets. *J Am Acad Child Adolesc Psychiatry*. 1988; 27(6): 726-737. doi:10.1097/00004583-198811000-00011
3. Boyce WT, Essex MJ, Woodward HR, Measelle JR, Ablow JC, Kupfer DJ. The confluence of mental, physical, social, and academic difficulties in middle childhood. I: exploring the "head waters" of early life morbidities. *J Am Acad Child Adolesc Psychiatry*. 2002; 41(5), 580-587. doi:10.1097/00004583-200205000-00016
4. Wan Y, Chen J, Sun Y, Tao F. Impact of childhood abuse on the risk of non-suicidal self-injury in mainland Chinese adolescents. *PLoS ONE* 2015; 10: e0131239. doi: 10.1371/journal.pone.0131239
5. Centers for Disease Control and Prevention (CDC). Adolescent and School Health: YRBSS Questionnaire 2013. CDC, 2013. (https://www.cdc.gov/healthyyouth/data/yrbs/ questionnaires.htm).
6. Wan Y, Chen R, Ma S, McFeeters D, Sun Y, Hao J, Tao F. Associations of adverse childhood experiences and social support with self-injurious behaviour and suicidality in adolescents. Br J Psychiatry. 2019; 214(3):146-152. doi: 10.1192/bjp.2018.263

© 2020 Zhang L et al. *JAMA Network Open.*
